# Chondrolysis of the Ankle Joint following Ankle Arthroscopy and Microfracture of the Osteochondral Lesion of the Talar Dome

**DOI:** 10.1155/2013/908082

**Published:** 2013-11-28

**Authors:** Tsz Lung Choi, Tun Hing Lui

**Affiliations:** ^1^Department of Orthopaedics and Traumatology, United Christian Hospital, Hong Kong; ^2^Department of Orthopaedics and Traumatology, North District Hospital, 9 Po Kin Road, Sheung Shui, Hong Kong

## Abstract

Chondrolysis of the ankle is a very rare condition. We report a case of chondrolysis of the ankle following ankle arthroscopy and microfracture of the osteochondral lesion of the talar dome. The patient's symptoms were relieved after articulated distraction arthroplasty.

## 1. Introduction

Chondrolysis is a clinical condition characterized by rapid destruction of articular cartilage on both sides of the joint leading to loss of joint space and joint stiffness. The cause has not truly been identified [1]. Chondrolysis has most commonly been described in the hip, shoulder, and knee joints. There were three cases of chondrolysis of the ankle joint reported in the English literature [1–3]. We report a case of chondrolysis of the ankle following ankle arthroscopy and microfracture of the osteochondral lesion of the talar dome.

## 2. Case Report

**Figure 1:**
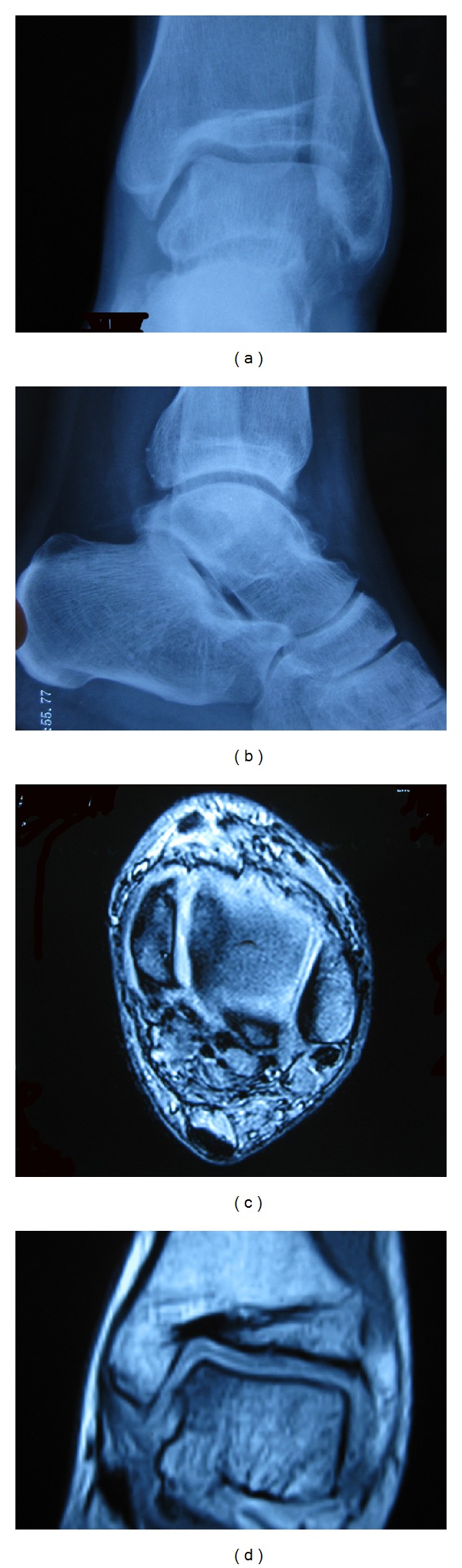
Anterolateral (a) and lateral (b) radiographs of the patient's left ankle before the ankle arthroscopy showed preservation of the joint space with mild anterior osteophytes. (c, d) magnetic resonance imaging showed the presence of osteochondral lesion of the medial talar dome.

**Figure 2:**
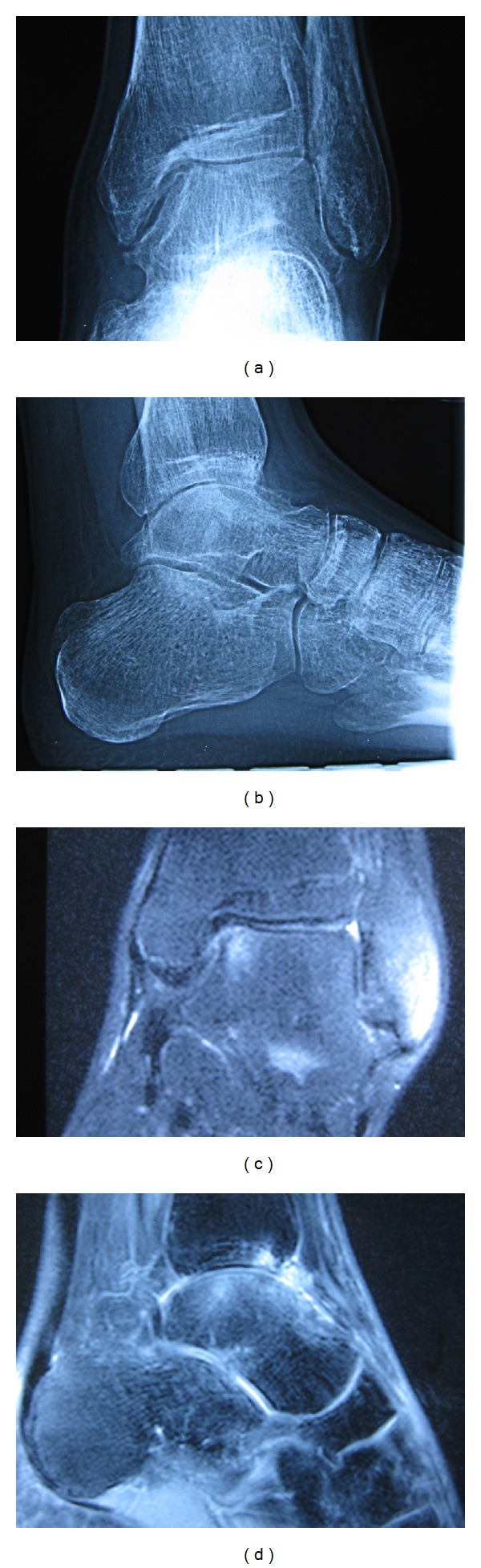
Radiographs (a, b) and magnetic resonance imaging (c, d) showed marked diffuse decrease in joint space of the ankle.

**Figure 3:**
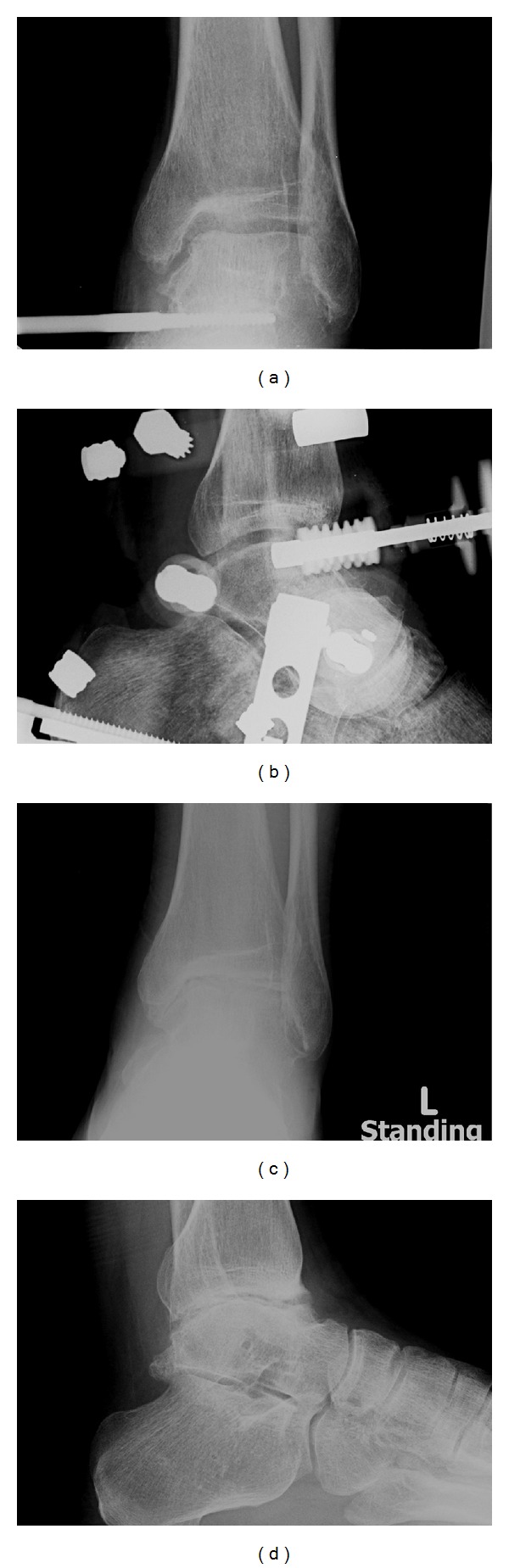
Anterolateral (a) and lateral (b) radiographs of the ankle with distraction arthroplasty showed creation of a space between the articular surfaces. (c, d) The ankle joint space remained diminished after removal of the external fixator.

A 32-year-old gentleman had inversion injury to his left ankle on 2007 resulting in persistent medial ankle pain. He was treated with physiotherapy without improvement. Radiographs and magnetic resonance imaging (MRI) of his left ankle showed the presence of osteochondral lesion (OCL) of the medial talar dome (Figure 1). Ankle arthroscopy and microfracture of the osteochondral lesion were performed on May 2008. His left ankle pain persisted after the operation and MRI of his left ankle was repeated and showed no interval change as compared to the previous MRI. Ankle arthroscopy was repeated on March 2009. Intraoperative findings showed that the medial talar OCL was covered with fibrocartilage and the other articular cartilage of the ankle joint was normal. However, his left ankle pain deteriorated afterwards as that he needed to walk with crutches. He complained of medial ankle pain and there was no rest pain or systemic upset. He was referred to our clinic for further management. Clinical examination showed that his left ankle was stiff with tenderness at the medial talar dome. Standing radiographs and MRI of his left ankle showed decreased ankle joint space without significant osteophytes formation, subchondral cyst, or subchondral sclerosis (Figure 2). Blood tests and gallium scan did not suggest any underlying infection. Ankle arthroscopy and distraction arthroplasty with Universal Compress Hinge were performed on May 2010. Arthroscopic findings showed marked fibrosis of the capsule with minimal synovitis. Most of the articular cartilage of the ankle joint was gone with exposure of the subchondral bone. There was an osseous defect at the medial talar dome. The patient was advised on active ankle mobilization exercise and weight bearing walking as pain was tolerated. The external fixator was removed three months later and his left ankle pain persisted with weight bearing. His left ankle pain started to improve 12 months after the distraction arthroplasty. During the latest followup on May 2012, he did not experience left ankle pain on walking although the ankle motion was still limited. Radiographs of his left ankle showed that the joint space remained decreased after removal of the external fixator (Figure 3).

## 3. Discussion

Chondrolysis is characterized by progressive loss of joint space and increased stiffness resulting from destruction of the articular cartilage on both sides of the joint [1]. It is a disabling condition that results in substantial pain and morbidity of the affected joint. It frequently affects those young and otherwise active patients [4].

The pain is usually out of proportion to the clinical findings, leading to a misdiagnosis of complex regional pain syndrome. This condition should be suspected in patients complaining of persistent, unrelenting pain, stiffness, and severe diffuse articular cartilage loss, within twelve months after an operation or potential cartilage insult to the ankle joint. The clinical symptoms should generally exceed a comparable amount of joint destruction in an otherwise chronic condition, for which the patient has likely had more time to adapt to the damage of the joint cartilage [4].

Proposed etiologic factors can be classified into four categories including (1) thermal (e.g., radiofrequency device, electrocautery, and holmium: YAG (yttrium-aluminum-garnet) laser), (2) chemical (e.g., intraarticular infusion of local anesthetics, chlorhexidine, and gentian violet), (3) mechanical (e.g., surgical insult or prominent hardware such as anchors, pins, or screws), or (4) other (e.g., infection or a history of traumatic joint injury) factors [4]. For the ankle, chemical toxicity due to infusion of local anesthetics [1] and mechanical insult [3] was noted as potential etiologic factor. To our knowledge, we describe the first case of idiopathic ankle chondrolysis after ankle arthroscopy and microfracture of the osteochondral lesion of the talar dome in the English literature.

Optimal treatment of chondrolysis of the ankle is inconclusive because of its rarity. Although total joint arthroplasty remains the gold standard for the treatment of extensive articular cartilage damage, nonarthroplasty options are reasonable for those with chondrolysis of the ankle as the patient is frequently of young age with high physical demands [4]. Nonoperative treatments include medication to decrease inflammation and pain and physiotherapy exercise under reduced load [1, 2]. This cannot relieve the pain in our patient and operative treatment was needed. Operative treatment of microfracture of the subchondral bone and joint lavage has also been used, but none of these gives prolonged improvement of the symptoms. Progression to complete destruction of the articular cartilage is expected and arthrodesis or replacement arthroplasty may be finally needed [2]. In this case, articulated distraction arthroplasty was chosen as the treatment choice because of the young age and relatively high physical demand of the patient. Articulated distraction creates a space between the bony surfaces, reduces mechanical stress, and provides ankle movement in order to restore the synovial circulation and encourage fibrous repair of the articular cartilage without the formation of adhesions [2]. This procedure has relieved the ankle pain experienced by our patient and deferred the need for arthrodesis or replacement arthroplasty.
